# 

**Published:** 1847-11

**Authors:** 


Brevet Col. McIntosh of the 5th Infantry, regular army, in his official report of the part which his troops took in the action of the 20th of August, when speaking of the march upon Cherubusco makes the following complimentary notice of an old friend and pupil of ours:

"Assistant Surgeon William Roberts accompanied the regiment during the march. His talent and zeal were not alone confined to his profession, but were displayed in a more military capacity, in aiding, assisting and urging on the men to the contest."

Roberts is a noble fellow, and we have no doubt has fairly earned this flattering notice from his superior.

*Private Hospital for the Insane at Chicago.*—Dr. Edward Mead, late Prof. of Materia Medica in the Medical Department of Illinois College, has opened at Chicago a private Hospital for the Insane.

